# Weighted Single-Step Genomic Evaluation of Body Structural Traits for Early Selection of Growth Performance in Thai Swamp Buffalo

**DOI:** 10.3390/ani16132012

**Published:** 2026-07-01

**Authors:** Wootichai Kenchaiwong, Vibuntita Chankitisakul, Monchai Duangjinda, Rawinan Lomngam, Kecha Kuha, Kitsanathon Sintala, Kulphat Pothikanit, Wuttigrai Boonkum

**Affiliations:** 1 Small Ruminant Research Unit, Faculty of Veterinary Science, Mahasarakham University, Mahasarakham 44000, Thailand; wootichai.k@msu.ac.th; 2 Network Center for Animal Breeding and Omics Research, Khon Kaen University, Khon Kaen 40002, Thailand; vibuch@kku.ac.th (V.C.); monchai@kku.ac.th (M.D.); 3 Department of Animal Science, Faculty of Agriculture, Khon Kaen University, Khon Kaen 40002, Thailand; lo_rawinan@kkumail.com; 4 Department of Animal Science, Faculty of Agriculture, Ubon Ratchathani Rajabhat University, Ubon Ratchathani 33000, Thailand; k.kuha3@gmail.com; 5 Department of Animal Science and Fisheries, Faculty of Sciences and Agricultural Technology, Rajamangala University of Technology Lanna, Nan 55000, Thailand; kitsanathon@rmutl.ac.th; 6 Buffalo Research and Development Group, Animal Breed Development Office, Department of Livestock Production, Bangkok 10400, Thailand; kulphatpothikanit@gmail.com

**Keywords:** type trait, growth trait, genetic parameter, genomic, GWAS, *Bubalus bubalis*

## Abstract

Early identification of genetically superior animals is important for improving growth performance and accelerating genetic gain in swamp buffalo breeding programs. This study investigated the genetic relationships between growth and body structural traits and evaluated their potential use as early selection indicators in Thai swamp buffalo. Phenotypic records from 1034 animals and genotypic data from 462 buffaloes genotyped with 30,979 SNP markers were analyzed using weighted single-step genomic best linear unbiased prediction (WssGBLUP) and weighted single-step Genome-Wide Association Study (WssGWAS) approaches. Moderate to high heritability estimates were observed for growth traits (0.41–0.59), whereas body structural traits showed low to moderate heritability (0.08–0.27). Positive genetic correlations were identified between growth traits and several structural traits, particularly heart girth, hip height, and body depth. Principal component analysis identified two major components explaining 80.1% of the total phenotypic variation, with the first principal component (PC1) representing overall body size and skeletal development. PC1 also showed relatively high heritability (0.57), indicating its potential utility as a composite selection trait. Genome-wide association analysis identified significant SNPs and candidate genes associated with weaning weight and PC1, including *ST6GALNAC5*, *EPHA6*, *SYN3*, *DDIT4*, *DNAJB12*, *BLCAP*, and *NNAT*, which are involved in growth regulation, metabolism, cellular development, and stress-response pathways. These findings demonstrate that body structural traits are genetically associated with growth performance and may serve as effective early selection indicators in genomic breeding programs for Thai swamp buffalo.

## Simple Summary

Swamp buffalo are important livestock in tropical regions, but genetic improvement is often limited by late-measured growth traits. This study evaluated whether early-life body structural traits could predict growth performance in Thai swamp buffalo using genomic analyses. Several body measurements related to body size showed favorable genetic associations with growth traits. Principal component analysis identified key components representing overall body structure, and genome-wide association analysis detected candidate genes related to growth and development. These findings indicate that body structural traits may serve as useful early selection indicators and could be incorporated into breeding programs to improve selection efficiency and accelerate genetic progress in Thai swamp buffalo.

## 1. Introduction

Improving productivity and efficiency in tropical livestock systems depends on selection strategies that facilitate the early and accurate identification of genetically superior animals, thereby accelerating genetic gain and enhancing production sustainability [1,2,3]. In swamp buffalo (*Bubalus bubalis*), a major livestock species in Southeast Asia, growth performance traits such as birth weight, weaning weight, and post-weaning body weight are economically important traits that strongly influence productivity, production efficiency, and profitability in buffalo production systems [4,5]. However, genetic improvement for these traits remains relatively slow due to long generation intervals and the late availability of phenotypic records, which delay the identification and selection of genetically superior animals [6,7,8]. These limitations emphasize the importance of identifying reliable early-life indicators to enhance selection efficiency and accelerate genetic progress in buffalo breeding programs.

Body structural traits have long been used in cattle breeding as indirect indicators of growth potential, functional performance, adaptability, and longevity [9]. Previous studies in dairy cattle have demonstrated significant genetic relationships between linear type traits and economically important traits, including milk production, fertility, body weight, and health traits [10]. These traits are generally measurable at an early age, moderately heritable, and biologically associated with skeletal development, muscular growth, and metabolic efficiency [11,12]. In ruminants, morphometric and structural traits—including body length, body depth, hip height, shoulder height, hip joint length, shoulder joint length, heart girth, fore shank circumference, fore knee circumference, hoof circumference, vulva width, and vulva length—show substantial genetic variability and moderate-to-strong inter-trait correlations, suggesting common genetic regulation of body size, skeletal growth, and functional conformation [13,14,15,16]. Moreover, multivariate analyses further indicate that these traits can be summarized into latent components representing general body size and reproductive-related morphology, suggesting their potential utility as composite indicators for selection [17].

Despite their potential utility, body structural traits have not yet been widely incorporated into buffalo breeding programs. Recent studies in dairy cattle have further demonstrated that linear type traits are genetically associated with functional, production, and reproductive performance and can therefore be incorporated into multi-trait selection strategies [18,19,20]. This represents a critical research gap, particularly in tropical production systems where early phenotyping is feasible but genomic information remains underutilized. Moreover, conventional pedigree-based models may not fully capture the genetic architecture of complex traits in buffalo populations, especially under heterogeneous environmental and management conditions [21,22].

Genomic selection offers an effective approach to overcome these limitations by increasing the accuracy of breeding value estimation and enabling the early selection of genetically superior animals [21,23,24]. Single-step genomic best linear unbiased prediction (ssGBLUP) combines pedigree, phenotypic, and genomic information within a unified framework, thereby improving the robustness and accuracy of genetic evaluation in populations with incomplete pedigree records or unbalanced datasets [22,25,26]. In addition, weighted single-step genomic best linear unbiased prediction (WssGBLUP) extends the ssGBLUP framework by assigning different weights to individual SNP markers according to their estimated contribution to genetic variance, thereby improving the detection of genomic regions associated with economically important traits and potentially enhancing prediction accuracy for complex traits controlled by loci with unequal effects [27]. Compared with conventional ssGBLUP, WssGBLUP has been widely applied in livestock species to improve genomic prediction and identify candidate genes underlying growth, reproductive, carcass, and adaptive traits [28,29,30]. Therefore, integrating WssGBLUP into genomic evaluation frameworks may provide additional advantages for genetic improvement programs, particularly for traits influenced by heat stress and environmental adaptation. Although genomic approaches have been extensively applied to growth and production traits in cattle, their application to body structural traits, particularly in relation to early prediction of growth performance in swamp buffalo, remains limited.

In buffalo populations, type traits have also been associated with production performance and have been proposed as useful indicators for the early selection of economically important traits [18,31,32,33,34,35]. In swamp buffalo, body-size-related traits generally exhibit moderate to high heritability estimates and contribute substantially to principal components associated with overall growth potential, skeletal development, and body conformation [14,17,19]. Furthermore, genome-wide association studies (GWAS) have identified several candidate genes associated with growth regulation, neuroendocrine signaling, and metabolic pathways, including *SYN3*, *CSRNP3*, and *NNAT*, supporting the biological relevance of integrating structural and growth traits in genomic evaluation programs [17,29,30,31]. Nevertheless, a comprehensive framework that integrates genomic evaluation of structural traits with their use as early predictors of growth performance has not yet been fully established in swamp buffalo populations.

Therefore, the objective of this study was to conduct a genomic evaluation of body structural traits and assess their potential for the early selection of growth performance in Thai swamp buffalo. Integrating genomic, phenotypic, and structural data provided novel insights into the genetic basis of body conformation and its relationship with growth performance, supporting the development of more efficient genomic breeding strategies for tropical buffalo populations.

## 2. Materials and Methods

All experimental procedures were approved by the Institutional Animal Care and Use Committee of Khon Kaen University (IACUC-KKU-24/67) and complied with the National Research Council of Thailand guidelines for animal experimentation.

### 2.1. Data Collection

**Figure 1:**
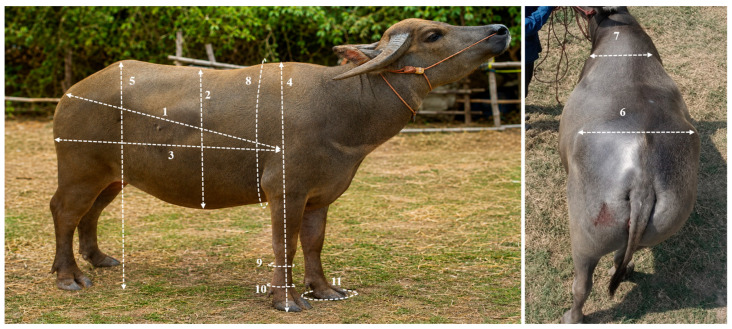
Body measurements: (1) BL = body length; (2) BD = body depth; (3) SL = shoulder length; (4) SH = shoulder height; (5) HH = hip height; (6) LH = length of hip joint; (7) LS = length of shoulder joint; (8) HG = heart girth; (9) FS = fore shank circumference; (10) FK = fore knee circumference; (11) HC = hoof circumference.

A total of 1034 phenotypic records were collected from Thai swamp buffaloes between 2019 and 2024 for growth traits, including birth weight (BW0), weaning weight at 240 days of age (WW240), and body weight at 400 days of age (BW400), as well as 11 structural traits: body length (BL), body depth (BD), shoulder length (SL), shoulder height (SH), hip height (HH), length of hip joint (LH), length of shoulder joint (LS), heart girth (HG), fore shank circumference (FS), fore knee circumference (FK), and hoof circumference traits (HC) (Figure 1). The pedigree dataset was comprised of 2843 animals across three generations, with 95.2% of individuals having complete sire and dam information. The dataset comprised animals maintained at five DLD buffalo breeding stations and 22 participating farmer herds distributed throughout Thailand’s major buffalo-producing areas, encompassing the northern, northeastern, central, eastern, and southern parts of the country.

Genotypic data were obtained from 474 Thai swamp buffaloes using the Axiom Buffalo 90K SNP Genotyping Array and the GeneTitan System (Thermo Fisher Scientific, Santa Clara, CA, USA). Animals were selected using stratified random sampling across high-, medium-, and low-EBV categories within both sexes to ensure representation across the breeding value distribution and to maximize genetic diversity among genotyped animals. Because genomic analyses were performed using the ssGBLUP/WssGBLUP/WssGWAS framework, which integrates phenotypic, pedigree, and genomic information from all available animals through the H matrix, potential bias associated with selective genotyping was expected to be substantially reduced compared with conventional GWAS approaches based solely on genotyped individuals. Quality control was applied to both animals and SNP markers using the following criteria: minor allele frequency (MAF) > 0.05 and SNP call rate ≥ 90%. Animals and SNPs that did not meet these thresholds were excluded from further analyses. Additional filtering steps removed animals with duplicate genotypes and SNPs exhibiting Mendelian inconsistencies. All quality control procedures and SNP analyses were conducted using the BLUPF90+ software package version 2.56 [36]. Following quality control, 462 animals and 30,979 SNPs remained for subsequent analyses. Descriptive statistics for the studied traits are presented in Table 1.

### 2.2. Genetic Parameter Estimation

Variance components for growth and structural traits were estimated using single-step genomic restricted maximum likelihood (ssGREML) approaches under a multivariate animal model. Variance components and genetic parameters, including heritability, genetic correlations, and phenotypic correlations, were estimated using the AIREMLF90 and BLUPF90+ programs developed by Misztal et al. [36]. The multivariate animal model was specified as follows:

y=Xb+Za+Mm+Wc+e (Model 1: for BW0, and WW240)

**y** = **Xb** + **Za** + **e** (Model 2: for BW400, body structural traits, PC1 and PC2)

where y was the vector of observations for growth, structural, PC1, and PC2 traits; b was the vector of fixed effects including herd–month–year of birth, sex, region; a was the vector of additive genetic effects assumed to be $a\sim N\left(0,A\sigma_{a}^{2}\right),$ where A was an additive genetic relationship matrix using pedigree information, and $\sigma_{a}^{2}$ was the direct additive genetic variance; m was the vector of maternal genetic effects assumed to be $m\sim N\left(0,A\sigma_{m}^{2}\right)$, where $\sigma_{m}^{2}$ was the maternal genetic variance; c was the vector of maternal permanent environmental effects assumed to be $c\sim N\left(0,I\sigma_{c}^{2}\right),$ where I was an identity matrix, and $\sigma_{c}^{2}$ was the permanent environmental variance; e was the vector of residual effects assumed to be $\varepsilon \sim N\left(0,I\sigma_{e}^{2}\right),$ where $\sigma_{e}^{2}$ was the residual variance; and X,Z,M, and W were incidence matrices relating observations to fixed, additive genetic effects, maternal genetic effects, and maternal permanent environmental effects, respectively. The variance–covariance structure was defined as follows:

$$Var\left[\begin{array}{c} a\\ m\\ c\\ \varepsilon \end{array}\right]=\left[\begin{array}{cccc} G_{0}⊗H & G_{0,am}⊗H & 0 & 0\\ G_{0,am}⊗H & M_{0}⊗H & 0 & 0\\ 0 & 0 & W_{0}⊗I_{c} & 0\\ 0 & 0 & 0 & R_{0}⊗I_{e} \end{array}\right]$$

where $G_{0}$, $M_{0}$, $W_{0}$, and $R_{0}$ were the additive genetic, maternal genetic, maternal permanent environmental, and residual (co)variance matrices among traits, respectively; H was the pedigree-genomic relationship matrix used in ssGREML; I was an identity matrix, and ⊗ denoted the Kronecker product. For pedigree-based analyses, the H matrix was replaced by A, the numerator relationship matrix.

### 2.3. Estimation of GEBVs

Genomic analyses were conducted using the weighted single-step genomic best linear unbiased prediction (WssGBLUP) method proposed by Wang et al. [27]. This approach integrates marker information by substituting the inverse pedigree relationship matrix ($A^{-1}$) in mixed model equations with $H^{-1}$ [36], as shown below:

$$H^{-1}=A^{-1}+\left[\begin{array}{cc} 0 & 0\\ 0 & {\tau G}^{-1}-{\omega A}_{22}^{-1} \end{array}\right]$$

where $A^{-1}$ was the inverse numerator relationship matrix for all animals; $G^{-1}$ was the inverse genomic relationship matrix for genotyped animals; and $A_{22}^{-1}$ was the inverse pedigree relationship matrix. Prior to the final genomic analyses, alternative scaling factors for the H matrix were evaluated following the recommendations of Aguilar et al. [21] and Wang et al. [27]. Several combinations of τ and ω were tested and assessed using prediction accuracy, regression coefficients of adjusted phenotypes on genomic estimated breeding values, and the extent of inflation or deflation in breeding value estimates. The combination τ = 1.00 and ω = 0.50 yielded the best overall performance, producing regression coefficients closest to unity and minimizing prediction bias. Consequently, this parameter set was selected for all subsequent analyses. The genomic relationship matrix (G) and SNP weights were constructed according to the method described by Lomngam et al. [8], as shown below:

$$G=\frac{{ZDZ}^{\prime }}{2\sum_{i=1}^{N}p_{i}(1-p_{i})}=\;{ZDZ}^{\prime }\varphi$$

where ***Z*** was a matrix of allele counts adjusted for allele frequencies (coded as 0, 1, or 2 for genotypes aa, Aa, and AA, respectively); D was a diagonal matrix of SNP-specific weights; N was the total number of SNP markers; $p_{i}$ was the minor allele frequency for the ith SNP; and φ represented the global scaling factor derived from $\varphi =\frac{\sigma_{u}^{2}}{\sigma_{a}^{2}}=\frac{1}{2\sum_{i=1}^{N}p_{i}(1-p_{i})}$. The algorithm then proceeded as described by Wang et al. [27] and Chen et al. [37].

### 2.4. Principal Component Analysis of Body Structural Traits

Principal component analysis (PCA) was performed to reduce the dimensionality of body structural traits and to identify the major sources of phenotypic variation in Thai swamp buffalo. The analyzed traits included body length, body depth, shoulder length, shoulder height, hip height, length of hip joint, length of shoulder joint, heart girth, fore shank circumference, fore knee circumference, and hoof circumference traits. Prior to PCA, body structural traits were adjusted using a general linear model with age and body weight fitted as covariates. The adjusted phenotypic values were then standardized to have a mean of zero and a standard deviation of one to eliminate scale differences among traits. Principal components (PC) were extracted from the correlation matrix using eigenvalue decomposition. Components with eigenvalues greater than 1.0 were retained according to the Kaiser criterion. Orthogonal varimax rotation was applied to improve the interpretability of the component structure. Traits with rotated component loadings greater than 0.50 were considered important contributors to a principal component. Uniqueness values were estimated to evaluate the proportion of variance not explained by the retained components. The retained principal component scores were subsequently used as composite traits in genetic parameter analyses.

### 2.5. Genome-Wide Association Study

The analyses were conducted using the weighted single-step Genome-Wide Association Study (WssGWAS) approach, following the procedures described by Wang et al. [38] and as previously applied in our earlier study [8]. Associations between quantitative traits and individual SNPs were estimated using the same mixed model framework as that applied in WssGBLUP. SNP effects were estimated through an iterative procedure implemented in the POSTGSF90 v. 1.83 software [36]. The additive genomic breeding value vector ($\hat{u}$) was transformed into SNP effects ($\hat{s}$) through their shared genomic variance $(\sigma_{u}^{2}$) as defined by the following equation:

$$\hat{s}=\;{DZ}^{\prime }G^{-1}\hat{u}$$

The additive genetic variance accounted for by each 10-SNP window was estimated using the following equation: $\frac{Var({\hat{s}}_{j})}{{\hat{\sigma }}_{u}^{2}}\times 100\%=\frac{Var(\sum_{k=j}^{j+9}z_{j}{\hat{s}}_{j})}{{\hat{\sigma }}_{u}^{2}}\;$, where ${Var(\hat{s}}_{j})\;$was the segment variance for the window starting at the jth SNP (10 consecutive SNPs); ${\hat{\sigma }}_{u}^{2}$ was the total additive genetic variance; $z_{j}$ was the vector of allele counts for the jth SNP across individuals; and ${\hat{s}}_{j}$ was the estimated effect of the jth SNP within the window. Significant SNPs were identified using a suggestive significance threshold of *p* ≤ 5 × 10^−6^ (−log_10_(*p*) ≥ 5.301). Manhattan plots, Quantile–Quantile (Q–Q) plots, and genomic inflation factors (λ) were generated using SAS Studio (version 3.81) to visualize GWAS results and assess the extent of test statistic inflation. Genomic inflation factors close to 1.0 indicated minimal population stratification and limited systematic bias.

### 2.6. Identification of Candidate and Pleiotropic Genes

Potential candidate genes for growth and structural traits were identified by searching genomic regions proximal to significant SNPs exceeding the variance threshold. Genes located within shared genomic regions across two or three traits were classified as pleiotropic. The maximum distance between candidate genes and SNP positions was set at 50 kb (kilobase pairs), consistent with previous studies [39]. Gene identification used the NCBI Genome Data Viewer (GDV) for the buffalo genome with UOA_WB_1 assembly [8,40] as reference. Further literature and database searches for all identified genes were conducted using the NCBI Gene and GeneCards platforms. For each gene, we recorded the search keywords, number of hits, and the database consulted.

## 3. Results

### 3.1. Genetic Parameters of Growth and Body Structural Traits

Estimated heritability, genetic correlations, and phenotypic correlations for growth and body structural traits in Thai swamp buffalo are presented in Table 1. Heritability estimates for growth traits in Thai swamp buffalo were moderate to high, ranging from 0.41 to 0.59. The highest heritability was observed for weaning weight at 240 days of age (WW240; 0.59), followed by birth weight (BW0; 0.52) and body weight at 400 days of age (BW400; 0.41). Body structural traits generally showed low to moderate heritability estimates, ranging from 0.08 to 0.27. Heart girth (HG; 0.27), length of hip joint (LH; 0.22), hoof circumference (HC; 0.19), hip height (HH; 0.16), and body depth (BD; 0.12) showed relatively higher heritability estimates compared with other structural traits (<0.10).

Genetic correlations among growth traits were positive, ranging from 0.17 to 0.88. The strongest genetic correlation was observed between WW240 and BW400 (0.88), whereas BW0 showed lower correlations with WW240 (0.37) and BW400 (0.17). Growth traits also showed positive genetic correlations with most body structural traits. WW240 exhibited moderate to high genetic correlations with all body structural traits (0.38 to 0.75). Similarly, BW400 showed positive genetic correlations with all body structural traits (0.33 to 0.70).

Phenotypic correlations among growth traits were positive and ranged from 0.23 to 0.78. The highest phenotypic correlation was found between WW240 and BW400 (0.78). Positive phenotypic correlations were also observed between growth and body structural traits, particularly for BL, BD, HH, and HG. Several structural traits showed strong positive relationships among themselves, including SH with LS (0.91), HH with SH (0.76), and HG with HH (0.84). Negative genetic correlations were observed for some traits involving hoof circumference and body length-related traits.

### 3.2. Principal Component of Body Structural Traits

**Figure 2:**
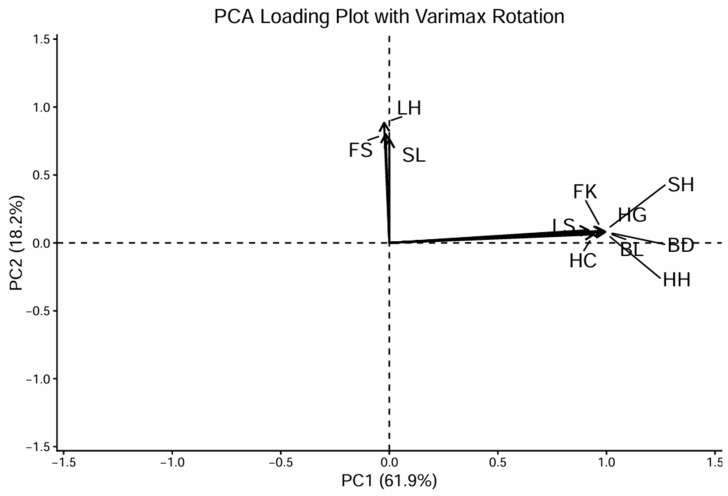
Principal component analysis (PCA) loading plot with Varimax rotation of body structural traits in Thai swamp buffalo. The first two principal components explained 80.1% of the total phenotypic variation, with PC1 and PC2 accounting for 61.9% and 18.2%, respectively.

Principal component analysis identified two principal components that together explained 80.1% of the total variation in body structural traits of Thai swamp buffalo (Figure 2 and Table 2). The first principal component (PC1) accounted for 61.9% of the total variance with an eigenvalue of 6.818, whereas the second principal component (PC2) explained 18.2% of the variance with an eigenvalue of 1.995. PC1 showed high positive loadings for most body size and skeletal traits, including hip height (0.994), heart girth (0.994), body length (0.991), body depth (0.985), fore knee circumference (0.986), length of shoulder joint (0.923), and hoof circumference (0.902). These traits generally exhibited low uniqueness values, ranging from 0.011 to 0.171. Shoulder height also contributed to PC1 with a moderate loading (0.467). PC2 was primarily associated with peripheral structural traits. High positive loadings were observed for length of hip joint (0.883), fore shank circumference (0.799) and shoulder length (0.762). The uniqueness values for these traits ranged from 0.219 to 0.417.

### 3.3. Genetic Parameters of Growth and Principal Components of Body Structural Traits

The variance components and genetic parameter estimate for growth traits and principal components of body structural traits in Thai swamp buffalo are presented in Table 3. Direct additive genetic variance (Va) was observed for all evaluated traits, ranging from 0.03 for PC1 to 425.76 for BW400. Residual variance (Ve) was highest for BW400 (656.15), followed by WW240 (98.48), whereas lower residual variances were observed for PC1 (0.02) and BW0 (4.44). Maternal genetic variance (Vm) and maternal permanent environmental variance (Vpe) were estimated only for BW0 and WW240. The maternal genetic variances were 1.58 for BW0 and 197.79 for WW240, while maternal permanent environmental variances were 1.78 and 5.96, respectively. Negative covariance between direct additive and maternal genetic effects (Vam) was observed for BW0 (−1.05) and WW240 (−194.91). Direct heritability estimates (h^2^) were moderate to high across traits, ranging from 0.30 to 0.58. The highest heritability estimate was observed for WW240 (0.58), followed by PC1 (0.57), BW0 (0.51), BW400 (0.39), and PC2 (0.30). Maternal heritability (m^2^) estimates were 0.10 for BW0 and 0.27 for WW240. The proportion of variance attributed to maternal permanent environmental effects (pe^2^) was 0.11 for BW0 and 0.01 for WW240.

### 3.4. Genome-Wide Association Analysis

**Figure 3:**
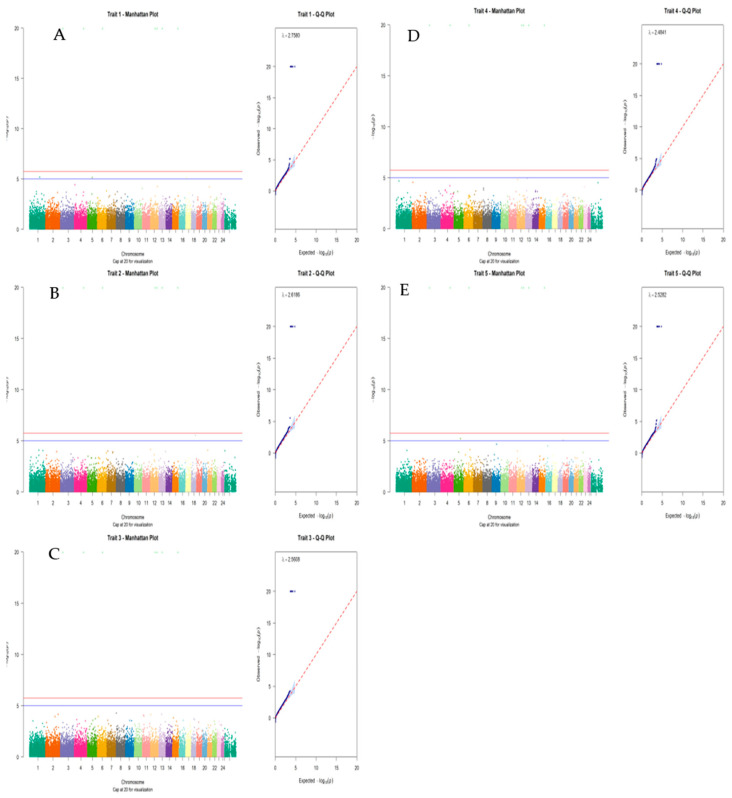
Manhattan and quantile–quantile (Q–Q) plots of SNP associations for (**A**) birth weight (BW0), (**B**) weaning weight at 240 days (WW240), (**C**) body weight at 400 days (BW400), (**D**) the first principal component (PC1), and (**E**) the second principal component (PC2) in Thai swamp buffalo. Manhattan plots show the genome-wide distribution of SNP associations expressed as −log_10_(*p*) values. The blue horizontal line indicates the suggestive significance threshold (*p* ≤ 5 × 10^−6^; −log_10_(*p*) = 5.301). Quantile–quantile (Q–Q) plots compare the observed and expected distributions of −log_10_(*p*) values to evaluate deviations from the null hypothesis of no association.

Manhattan and quantile–quantile (Q–Q) plots of SNP effects for direct genetic effects across all traits are shown in Figure 3. The genome-wide association analysis identified eight significant SNPs associated with weaning weight at 240 days of age (WW240) and the first principal component of body structural traits (PC1), as presented in Table 4. The WssGWAS analysis identified significant SNPs associated with growth and body structural traits based on SNP-level association statistics. SNPs exceeding the suggestive significance threshold of *p* ≤ 5 × 10^−6^ (−log_10_(*p*) ≥ 5.301) were considered significant and used for the identification of biologically relevant candidate genes and genomic regions. Among these, seven SNPs were associated with WW, whereas one SNP was associated with PC1. The identified SNPs were distributed across chromosomes 1, 2, 4, 5, 6, and 14. For WW, the most significant SNP was *AX-85180545* located on chromosome 6 at 67,147,933 bp (−log_10_(*p*) = 6.870), which was mapped within the *ST6GALNAC5* gene. Additional significant SNPs associated with WW included *AX-85103615* on chromosome 4 within *SYN3* (−log_10_(*p*) = 6.316), *AX-85076131* on chromosome 1 within *EPHA6* (−log_10_(*p*) = 6.223), and *AX-85141348* on chromosome 14 near *HCK*, *CCM2L*, and *XKR7* (−log_10_(*p*) = 5.783). Significant associations were also detected for *AX-85152233* near *MRGPRX2* on chromosome 5 and AX-85148405 near *ASCC1*, *ANAPC16*, *DDIT4*, and *DNAJB12* on chromosome 4. In addition, SNP *AX-85117485* on chromosome 2 was located near *CSRNP3* and *SCN2A*. For body structural traits, one significant SNP (*AX-85045075*) associated with PC1 was identified on chromosome 14 at 17,123,254 bp (−log_10_(*p*) = 5.473). This SNP was located near *BLCAP* and *NNAT*.

## 4. Discussion

This study provided a comprehensive genomic evaluation of body structural traits and demonstrated their potential utility for the early selection of growth performance in Thai swamp buffalo. The results indicated that several structural traits were under moderate genetic control and were strongly correlated, reflecting a shared biological basis related to body size and skeletal development. Moderate to high heritability estimates for key structural traits suggested that these phenotypes could respond effectively to selection and serve as reliable indicators in breeding programs. Similarly, the moderate to high heritability estimates observed for growth traits indicated substantial additive genetic variation, suggesting that these traits could also respond favorably to genetic selection [8,41,42,43]. In particular, the high heritability estimates for weaning weight at 240 days of age (0.59) and birth weight (0.52) demonstrate the potential for genetic improvement through selective breeding in Thai swamp buffalo populations. Similar moderate to high heritability estimates for growth traits have been reported in other buffalo populations, indicating that body weight traits are largely under genetic control [7,44,45]. Body structural traits showed low to moderate heritability estimates, ranging from 0.08 to 0.27, consistent with previous studies in buffalo and beef cattle reporting moderate genetic influences on morphological and skeletal traits [46,47,48]. Among these traits, heart girth, hip joint length, and hoof circumference exhibited relatively higher heritability estimates, suggesting their potential utility as indirect selection criteria for growth performance. Heart girth is strongly associated with body capacity, skeletal development, and live weight estimation in livestock, including buffaloes [49,50]. The relatively lower heritability estimates observed for several structural traits may be attributed to environmental sensitivity, measurement variability, and the complex polygenic nature of skeletal development.

The positive genetic correlations observed among growth traits suggested that selection for superior early growth performance could simultaneously improve later growth characteristics [44]. In particular, the strong genetic correlation between weaning weight at 240 days (WW240) and body weight at 400 days (BW400) (0.88) indicates that weaning weight is an effective early predictor of subsequent growth potential [51]. In contrast, the lower genetic correlations involving birth weight suggest that prenatal and postnatal growth may be partially regulated by different physiological and genetic mechanisms [41,52]. This finding was biologically plausible because birth weight was strongly influenced by the maternal uterine environment and fetal development, whereas postnatal growth increasingly depends on nutrient utilization and metabolic efficiency [53]. The positive genetic and phenotypic correlations observed between growth and body structural traits further support the biological integration of body development and skeletal growth [54,55]. Relationships between body conformation traits and productive performance have been reported in dairy buffaloes, where type traits were proposed as valuable indicators for early selection of milk production potential [34]. Similar associations have been reported in buffalo, cattle, and other livestock species, in which body conformation traits are associated with muscularity, residual feed intake, metabolic efficiency, and productive performance [56,57]. The strong phenotypic associations among shoulder height, shoulder length, and hip height also suggest coordinated skeletal development and structural proportionality. In contrast, several body length-related traits exhibited negative genetic correlations with pelvic and distal skeletal measurements, particularly between body length and length of hip joint (−0.41). This relationship may reflect differential genetic regulation of trunk and pelvic development. Body length primarily represents longitudinal growth of the vertebral column and overall frame size, whereas hip joint length reflects pelvic morphology and hindquarter structure. Consequently, genetic factors promoting body elongation may not necessarily result in proportional enlargement of pelvic dimensions. Furthermore, skeletal growth in livestock often follows allometric patterns, whereby different anatomical regions develop at different rates and are influenced by partially distinct genetic pathways. From a functional perspective, more compact pelvic structures in longer-bodied animals may also contribute to maintaining biomechanical balance, locomotor efficiency, and effective weight distribution. Therefore, the observed antagonistic relationship likely reflects developmental trade-offs among skeletal regions rather than adverse biological effects.

Principal component analysis (PCA) revealed that two principal components explained 80.1% of the total variation in body structural traits, indicating that body conformation in Thai swamp buffalo can be effectively summarized using a reduced number of biologically meaningful variables. The first principal component (PC1), which accounted for 61.9% of the total variance, was strongly associated with body size and skeletal development traits, including hip height, heart girth, body length, body depth, and fore knee circumference. These results suggest that PC1 primarily represents overall body size and structural development. Similar findings have been reported in previous morphometric studies, in which the first principal component generally reflects overall body size and growth-related morphological variation [17,58]. The high factor loadings and low uniqueness values observed for these traits indicate substantial shared variation and further support the effectiveness of PCA for dimensional reduction in livestock breeding studies. The second principal component (PC2) was mainly associated with length of hip joint dimension, shoulder length, and fore shank circumference, suggesting that this component represents peripheral structural characteristics. These findings suggest that incorporating principal components into selection strategies may improve breeding efficiency by targeting composite biological functions rather than individual traits alone [59,60]. Moreover, the moderate to high heritability estimates observed for PC1 (0.57) and PC2 (0.30) indicate that these composite traits are under genetic control and may serve as effective selection indices [61]. The relatively high heritability estimate observed for PC1 (0.57) compared with several individual structural traits may initially appear unexpected. However, PC1 represents a latent composite trait derived from multiple highly correlated body measurements that collectively describe overall body size and skeletal development. Because principal component analysis concentrates the variation shared among correlated traits while reducing trait-specific environmental noise and measurement error, the resulting component may exhibit a larger proportion of additive genetic variance relative to total phenotypic variance. Similar increases in heritability for principal component-derived traits have been reported in livestock populations, where composite traits capture common biological processes more effectively than individual measurements. Therefore, the high heritability of PC1 suggests that overall body conformation may be under stronger genetic control than indicated by any single structural trait alone.

Genome-wide association approaches have successfully identified genomic regions associated with body linear type traits in dairy cattle, supporting the effectiveness of genome-wide analyses for dissecting the genetic architecture of body conformation traits [62]. Among the identified loci, *ST6GALNAC5*, *EPHA6*, *SYN3*, and *DDIT4* are particularly notable because they are involved in pathways related to cell proliferation and metastasis (*ST6GALNAC5*) [63,64], neural development and synaptic signaling (*EPHA6* and *SYN3*) [65,66], and metabolic and stress-response regulation through mTOR signaling (*DDIT4*) [67]. The identification of *DDIT4* and *DNAJB12* within the genomic region associated with WW240 was noteworthy, as both genes were involved in cellular stress-response pathways that may influence growth under tropical production conditions. *DDIT4* (*REDD1*) is a stress-inducible regulator of the mTOR signaling pathway that controls cell growth, protein synthesis, energy metabolism, and responses to hypoxia and nutrient deprivation. Therefore, genetic variation near *DDIT4* may affect resource allocation between growth and adaptation during the weaning period. *DNAJB12*, a member of the Hsp40/DnaJ chaperone family, contributes to protein quality control, endoplasmic reticulum homeostasis, and the degradation of misfolded proteins. Its proximity to a WW240-associated SNP suggests that protein homeostasis may contribute to growth resilience in tropical environments. However, these genes were considered biologically plausible positional candidates rather than confirmed causal genes. Further functional studies, including gene expression analyses under heat or nutritional stress, are needed to elucidate their roles in buffalo growth and adaptation. Similarly, *EPHA6* and *SYN3* participate in neural and developmental signaling pathways that may indirectly influence growth regulation and physiological adaptation. Candidate genes associated with PC1, including *BLCAP* and *NNAT*, are involved in apoptosis, metabolic homeostasis, and growth regulation. Previous studies have associated neuronatin (*NNAT*) with growth traits, adipocyte differentiation, lipid deposition, and energy metabolism in livestock species [68,69]. These findings supported the hypothesis that body structural development was regulated by complex molecular pathways involving metabolism, cellular growth, and endocrine signaling. In addition, genome-wide association studies in cattle have identified significant genomic regions associated with stature and body size traits, further supporting the polygenic nature of structural development and growth-related phenotypes [70]. Furthermore, the identification of multiple candidate genes involved in stress response, immune regulation, and developmental processes highlights the polygenic nature of growth and structural traits in buffalo populations. In contrast, genes including *HCK*, *CCM2L*, *XKR7*, *MRGPRX2*, *ASCC1*, *ANAPC16*, and *SCN2A* had limited direct evidence linking them to growth performance or body conformation in livestock species. Nevertheless, several of these genes participated in biological processes that may indirectly influence growth and development. For example, *HCK* and *MRGPRX2* are involved in immune regulation, *CCM2L* contributes to angiogenesis and vascular integrity, *XKR7* is associated with apoptosis and membrane remodeling, *ASCC1* participates in transcriptional regulation and DNA repair, *ANAPC16* plays a role in cell-cycle regulation, and *SCN2A* is involved in neural signaling. These biological functions may affect growth and body development through their influence on tissue growth, cellular homeostasis, and developmental pathways [71,72]. Therefore, these genes should be considered positional candidate genes located within or near significant genomic regions rather than confirmed causal genes. Similar observations have been reported in previous GWASs of livestock growth and body-size traits, in which numerous positional candidate genes were identified, but additional functional validation was required to establish their biological significance [73,74]. Additional functional genomics, transcriptomic, and gene-expression studies will be required to clarify the biological roles of these genes and determine their potential contributions to growth and body structural traits in Thai swamp buffalo.

However, despite the strong genetic correlation between WW240 and BW400 and the substantial additive genetic variance estimated for BW400, no SNP window exceeded the predefined significance threshold in the WssGWAS analysis. Although WW240 and BW400 shared a high genetic correlation (0.88), this relationship was not complete, indicating that a proportion of the genetic variation affecting BW400 is distinct from that influencing WW240. Consequently, genomic regions associated with WW240 may not necessarily remain detectable at 400 days of age. The absence of significant SNP windows for BW400 therefore does not imply a lack of genetic determinism. Instead, it may suggest that BW400 is controlled by a highly polygenic architecture, in which numerous loci with small effects contribute to phenotypic variation, reducing the likelihood that any individual genomic window explains enough additive genetic variance to reach the significance threshold. From a genomic perspective, the application of weighted single-step genomic best linear unbiased prediction (WssGBLUP) enabled more accurate estimation of genetic parameters by simultaneously integrating pedigree and genomic information. The integration of structural traits with genomic information may enhance early selection strategies in swamp buffalo. Unlike growth traits, structural traits can be measured earlier in life, enabling earlier identification of genetically superior animals. This is particularly valuable in buffalo production systems characterized by long generation intervals and limited recording infrastructure.

The positive genetic correlations between structural and growth traits suggested that structural traits could serve as early indicator traits in genomic selection programs, thereby improving breeding efficiency. Strong genetic relationships among size-related traits also support the use of selection indices that combine growth and structural traits [75]. Selection based on composite traits, such as PC1, may improve overall selection accuracy while reducing the effects of environmental variation and measurement error. This approach is consistent with modern multi-trait breeding strategies aimed at improving robustness, adaptability, and sustainable production. However, several limitations should be considered. Genetic relationships may be influenced by population structure and environmental heterogeneity commonly observed in tropical production systems. Further studies are needed to evaluate genotype-by-environment interactions and to validate principal component traits through functional and longitudinal analyses. In addition, increasing population size and SNP density would likely improve genomic prediction accuracy and the statistical power of genome-wide association studies (GWAS). Although genotyped animals were selected using a stratified sampling strategy based on EBV categories, the potential impact of selective genotyping was minimized by the application of the single-step genomic framework. Unlike conventional GWAS approaches that rely exclusively on genotyped animals, WssGWAS utilizes information from the entire pedigree, phenotypic, and genomic dataset simultaneously. Consequently, SNP effect estimation is informed by all available animals in the population, reducing the risk of false-positive associations arising from allele-frequency differences among selectively genotyped groups. An additional limitation relates to the pedigree structure used in the single-step genomic evaluation. Although the pedigree dataset exhibited a high level of completeness, with 95.2% of animals having known sire and dam information, variations in pedigree depth among individuals may still influence the construction of the relationship matrix and the blending of pedigree and genomic information. Future studies should incorporate formal pedigree quality metrics, such as pedigree completeness index and equivalent complete generations, to further assess the potential impact of pedigree structure on genomic prediction accuracy and breeding value estimation.

## 5. Conclusions

This study provided insights into the genetic and genomic relationships between body structural and growth traits in Thai swamp buffalo. Moderate to high heritability estimates and favorable genetic correlations indicated that body structural traits may be useful as early selection indicators for growth performance. Principal component analysis further demonstrated the effectiveness of multivariate approaches for summarizing body conformation traits related to overall body size and morphology. In addition, genome-wide association analysis identified candidate genes associated with growth, metabolism, and cellular development, supporting the polygenic nature of these traits. These findings provided a genomic basis for incorporating body structural traits into breeding programs to improve growth performance in Thai swamp buffalo.

## Figures and Tables

**Table 1:** Heritability (diagonal), genetic correlations (upper diagonal), and phenotypic correlations (lower diagonal) for growth and body structural traits in Thai swamp buffalo.

Traits	BW0	WW240	BW400	BL	BD	SL	SH	HH	LH	LS	HG	FS	FK	HC
BW0	0.52	0.37	0.17	0.25	0.22	0.18	0.20	0.24	0.21	0.19	0.30	0.16	0.18	0.10
WW240	0.27	0.59	0.88	0.65	0.60	0.52	0.58	0.62	0.55	0.53	0.75	0.48	0.51	0.38
BW400	0.23	0.78	0.41	0.60	0.55	0.45	0.50	0.58	0.48	0.46	0.70	0.40	0.44	0.30
BL	0.18	0.42	0.45	0.08	0.25	0.30	0.07	0.14	−0.41	−0.08	0.11	−0.24	−0.16	−0.23
BD	0.16	0.38	0.42	0.12	0.12	0.30	0.28	0.36	0.24	0.26	0.52	0.20	0.22	0.12
SL	0.12	0.32	0.35	0.10	0.18	0.10	0.62	0.57	0.52	0.65	0.50	0.48	0.55	0.05
SH	0.15	0.36	0.38	0.04	0.08	0.25	0.09	0.50	0.14	0.95	0.68	0.16	0.93	−0.11
HH	0.17	0.40	0.44	0.19	0.12	0.30	0.76	0.16	0.00	0.78	0.93	0.12	0.76	0.13
LH	0.14	0.34	0.36	0.03	0.09	0.22	0.42	0.18	0.22	0.33	0.12	0.84	0.22	−0.03
LS	0.13	0.33	0.35	0.08	0.10	0.28	0.91	0.58	0.36	0.12	0.75	0.35	0.96	0.00
HG	0.22	0.50	0.55	0.15	0.20	0.24	0.41	0.84	0.16	0.48	0.27	0.27	0.74	0.28
FS	0.10	0.28	0.30	0.02	0.06	0.18	0.58	0.28	0.68	0.50	0.23	0.13	0.19	−0.04
FK	0.12	0.30	0.32	0.07	0.08	0.20	0.87	0.60	0.34	0.08	0.49	0.49	0.14	0.15
HC	0.05	0.20	0.22	−0.12	0.03	0.05	−0.02	−0.02	−0.02	−0.01	−0.01	−0.06	−0.01	0.19

BW0 = birth weight; WW240 = weaning weight at 240 days of age; BW400 = body weight at 400 days of age; BL = body length; BD = body depth; SL = shoulder length; SH = shoulder height; HH = hip height; LH = length of hip joint; LS = length of shoulder joint; HG = heart girth; FS = fore shank circumference; FK = fore knee circumference; HC = hoof circumference.

**Table 2:** Principal component (PC) loadings, uniqueness, and variance explained for body structural traits in Thai swamp buffalo.

Traits	PC1	PC2	Uniqueness
BL	0.991		0.018
BD	0.985		0.030
SH	0.467		0.776
HH	0.994		0.012
HC	0.910		0.171
LS	0.923		0.147
HG	0.994		0.011
FK	0.986		0.027
SL		0.762	0.417
FS		0.799	0.361
LH		0.883	0.219
Variance explained by Principal components	Eigenvalue	Proportion of variance	Cumulative variance
PC1	6.818	0.619	0.619
PC2	1.995	0.182	0.801

BL = body length; BD = body depth; SL = shoulder length; SH = shoulder height; HH = hip height; LH = length of hip joint; LS = length of shoulder joint; HG = heart girth; FS = fore shank circumference; FK = fore knee circumference; HC = hoof circumference.

**Table 3:** Variance components and genetic parameter (heritability, maternal, and permanent environmental effects) for growth and principle component (PC) of body structural traits in Thai swamp buffalo.

Parameter/Traits	BW0	WW240	BW400	PC1	PC2
Va	8.08	422.26	425.76	0.03	0.35
Vam	−1.05	−194.91	-	-	-
Vm	1.58	197.79	-	-	-
Vpe	1.78	5.96	-	-	-
Ve	4.44	98.48	656.15	0.02	0.84
h^2^	0.51	0.58	0.39	0.57	0.30
m^2^	0.10	0.27	-	-	-
pe^2^	0.11	0.01	-	-	-

Va = direct additive variance; Vm = maternal variance; Vam = additive and maternal covariance; Vpe = maternal permanent environmental variance; Ve = residual vari-ance, h^2^ = direct additive heritability; m^2^ = maternal heritability; pe^2^ = ratio of vari-ance due to maternal permanent environmental effect to total phenotypic variance. BW0 = birth weight; WW240 = weaning weight at 240 days of age; BW400 = body weight at 400 days of age; PC1 = principal component 1; PC2 = principal component 2.

**Table 4:** Significant SNPs and candidate genes associated with growth and principal component of body structural traits identified by genome-wide association analysis in Thai swamp buffalo.

No.	SNP Name	*p*-Value	Chromosome	Location (bp)	Gene	Distance to SNP (bp)	Traits	Putative Function
1	AX-85103615	6.316	4	48707789	*SYN3*	0	WW240	Synaptic signaling, neuronal development, and neuroendocrine regulation
2	AX-85117485	5.381	2	83339639	*CSRNP3*	27,428	WW240	Gene expression regulation, cell differentiation, and apoptosis regulation
					*SCN2A*	−39,391	WW240	Ion transport, neural signaling, and neurophysiological regulation
3	AX-85045075	5.473	14	17123254	*BLCAP*	−10,817	PC1	Apoptosis signaling, post-transcriptional regulation, and cellular homeostasis
					*NNAT*	−19,747	PC1	Calcium-mediated signaling, growth regulation, and metabolic homeostasis
4	AX-85180545	6.870	6	67147933	*ST6GALNAC5*	0	WW240	Glycoprotein modification, cell adhesion, and signal transduction
5	AX-85076131	6.223	1	86609827	*EPHA6*	0	WW240	Cell signaling, tissue development, and cell migration regulation
6	AX-85141348	5.783	14	22013609	*HCK*	6350	WW240	Signal transduction, immune function, and cellular growth regulation
					*CCM2L*	−12,796	WW240	Signal transduction, angiogenesis, and tissue integrity regulation
					*XKR7*	−39,572	WW240	Membrane remodeling, apoptosis regulation, and cellular homeostasis
7	AX-85152233	5.687	5	101555810	*MRGPRX2*	1278	WW240	G protein-coupled receptor signaling, immune response activation, and inflammatory regulation
8	AX-85148405	5.527	4	147655598	*ASCC1*	43,201	WW240	Gene expression regulation, DNA repair, and cellular development
					*ANAPC16*	33,284	WW240	Cell cycle control, protein degradation, and cellular proliferation
					*DDIT4*	5759	WW240	Stress signaling, metabolic regulation, and growth suppression
					*DNAJB12*	−35,181	WW240	Molecular chaperone activity, heat stress response, and protein quality control

*SYN3:* Synapsin III, *CSRNP3:* Cysteine and serine rich nuclear protein 3, *SCN2A:* Sodium voltage-gated channel alpha subunit 2, *BLCAP:* Bladder cancer-associated protein (apoptosis-inducing factor), *NNAT:* Neuronatin. *ST6GALNAC5:* ST6 N-acetylgalactosaminide alpha-2,6-sialyltransferase 5, *EPHA6:* EPH receptor A6, *HCK:* HCK proto-oncogene, Src family tyrosine kinase, *CCM2L*: CCM2-like scaffold protein, *XKR7*: XK-related protein 7, *MRGPRX2*: Mas-related G protein-coupled receptor member X2, *ASCC1*: Activating signal cointegrator 1 complex subunit 1, *ANAPC16*: Anaphase-promoting complex subunit 16, *DDIT4*: DNA damage-inducible transcript 4, *DNAJB12*: DNAJ heat shock protein family (Hsp40) member B12.

## Data Availability

The original contributions presented in this study are included in this article. Further inquiries can be directed to the corresponding author.
